# Novel Functional Properties of Missense Mutations in the Glycine Receptor β Subunit in Startle Disease

**DOI:** 10.3389/fnmol.2021.745275

**Published:** 2021-09-24

**Authors:** Inken Piro, Anna-Lena Eckes, Vikram Babu Kasaragod, Claudia Sommer, Robert J. Harvey, Natascha Schaefer, Carmen Villmann

**Affiliations:** ^1^Department of Neurology, University Hospital Würzburg, Würzburg, Germany; ^2^Institute for Clinical Neurobiology, University Hospital, Julius-Maximilians-University Würzburg, Würzburg, Germany; ^3^Neurobiology Division, MRC Laboratory of Molecular Biology, Cambridge, United Kingdom; ^4^School of Health and Behavioural Sciences, University of the Sunshine Coast, Maroochydore, QLD, Australia; ^5^Sunshine Coast Health Institute, Birtinya, QLD, Australia

**Keywords:** *GLRB*, glycine receptor, hyperekplexia, startle disease, gephyrin

## Abstract

Startle disease is a rare disorder associated with mutations in *GLRA1* and *GLRB*, encoding glycine receptor (GlyR) α1 and β subunits, which enable fast synaptic inhibitory transmission in the spinal cord and brainstem. The GlyR β subunit is important for synaptic localization via interactions with gephyrin and contributes to agonist binding and ion channel conductance. Here, we have studied three *GLRB* missense mutations, Y252S, S321F, and A455P, identified in startle disease patients. For Y252S in M1 a disrupted stacking interaction with surrounding aromatic residues in M3 and M4 is suggested which is accompanied by an increased EC_50_ value. By contrast, S321F in M3 might stabilize stacking interactions with aromatic residues in M1 and M4. No significant differences in glycine potency or efficacy were observed for S321F. The A455P variant was not predicted to impact on subunit folding but surprisingly displayed increased maximal currents which were not accompanied by enhanced surface expression, suggesting that A455P is a gain-of-function mutation. All three GlyR β variants are trafficked effectively with the α1 subunit through intracellular compartments and inserted into the cellular membrane. *In vivo*, the GlyR β subunit is transported together with α1 and the scaffolding protein gephyrin to synaptic sites. The interaction of these proteins was studied using eGFP-gephyrin, forming cytosolic aggregates in non-neuronal cells. eGFP-gephyrin and β subunit co-expression resulted in the recruitment of both wild-type and mutant GlyR β subunits to gephyrin aggregates. However, a significantly lower number of GlyR β aggregates was observed for Y252S, while for mutants S321F and A455P, the area and the perimeter of GlyR β subunit aggregates was increased in comparison to wild-type β. Transfection of hippocampal neurons confirmed differences in GlyR-gephyrin clustering with Y252S and A455P, leading to a significant reduction in GlyR β-positive synapses. Although none of the mutations studied is directly located within the gephyrin-binding motif in the GlyR β M3-M4 loop, we suggest that structural changes within the GlyR β subunit result in differences in GlyR β-gephyrin interactions. Hence, we conclude that loss- or gain-of-function, or alterations in synaptic GlyR clustering may underlie disease pathology in startle disease patients carrying *GLRB* mutations.

## Introduction

Glycine receptors (GlyRs) enable fast synaptic inhibition in the adult brainstem and spinal cord of rodents and humans. In addition, GlyRs have been detected in the adult organism in the cortex, the retina, and inner ear (Lynch, 2004). GlyRs belong to the cys-loop ligand-gated ion channel superfamily that includes nicotinic acetylcholine receptors, GABA_A/C_ receptors, and 5HT_3_ receptors. Cys-loop receptors are pentamers with a large N-terminal domain (NTD) composed of an N-terminal α-helix followed by 10 β-strands forming a twisted β-sheet arrangement, providing an immunoglobulin-like fold (Du et al., 2015; Huang et al., 2015; Yu et al., 2021). All receptor subunits have four transmembrane domains (TMD) followed by a short extracellular C-terminus. Transmembrane segments 1–4 (M1–M4) are connected by two small loops (M1–2 loop and M2–3 loop) and a large intracellular loop between M3 and M4 (Lynch, 2004). This loop is part of the intracellular domain (ICD) and of highest diversity among the subunits harboring specific domains for protein-protein interactions (Langlhofer and Villmann, 2016).

For GlyRs, four α subunits and one β subunit have been identified. GlyR α subunits can form functional homomeric channels located at extrasynaptic and presynaptic sites (Turecek and Trussell, 2001; Xiong et al., 2014). By contrast, β subunits only form functional ion channels when co-assembled with α subunits in heteromeric receptor complexes (Bormann et al., 1993). The subunit stoichiometry of heteromeric GlyRs has been described as 3α:2β pentameric assemblies (Durisic et al., 2012; Patrizio et al., 2017). During embryonic development, homomeric α2 subunit GlyRs represent the major GlyR isoform whereas after birth, subunit switches result in α1β and α3β as the major GlyR isoforms at postsynaptic sites (Liu and Wong-Riley, 2013; Morelli et al., 2017). Homomeric and heteromeric GlyRs also differ in their agonist and antagonist affinities (Grudzinska et al., 2005). Furthermore, Pribilla et al. (1992) demonstrated significant differences in the inhibition of the homomeric versus heteromeric GlyRs by the plant alkaloid picrotoxin. While homomeric α subunit GlyRs are blocked by picrotoxin, heteromeric αβ channels are almost unaffected (50–200-fold less effective) by the toxin (Pribilla et al., 1992).

During protein maturation, GlyR complexes fold and traffic through cellular compartments including the endoplasmic reticulum (ER), the ER-Golgi intermediate compartment (ERGIC) and the Golgi apparatus, with misfolded receptors retained in the ER (Schaefer et al., 2015, 2018). In neurons and transfected cells, α and β subunits are transported together with the scaffold protein gephyrin in a complex toward the neuronal membrane (Meyer et al., 1995; Meier et al., 2000; Patrizio et al., 2017). Residues 391–408 (numbering refers to precursor protein) localized in the M3-M4 loop of the GlyR β subunit enable gephyrin binding (Meyer et al., 1995; Maric et al., 2017).

Disruption of glycinergic neurotransmission is associated with neurological disorders including startle disease (hyperekplexia, OMIM 147100) and stiff person syndrome (SPS). The majority of cases of startle are caused by genetic variants in *GLRA1* and *GLRB* (encoding the GlyR α1 and β subunits) or *SLC6A5* encoding the glycine transporter 2 (GlyT2) (Rees et al., 2006; Chung et al., 2010, 2013; James et al., 2013; Bode and Lynch, 2014; Schaefer et al., 2015). By contrast, SPS patients suffer from impaired inhibitory neurotransmission following binding of GlyR-autoantibodies to the GlyR NTD (Carvajal-Gonzalez et al., 2014; Rauschenberger et al., 2020).

Genetic variants in *GLRA1* and *GLRB* are inherited in either a dominant or recessive manner. Dominant mutations mainly affect ion channel function, e.g., ligand binding or channel opening, while most recessive mutations result in protein trafficking deficits (Villmann et al., 2009; Chung et al., 2010; Bode and Lynch, 2014; Schaefer et al., 2015). Mutations in the *GLRB* gene represent the third most common cause for startle disease. Homozygous null mutations including nonsense, small indels, frameshifts and splicing variants have been described. Moreover, missense mutations P169L, M177R, L285R, W310C, and Y470C located in the extracellular domain (ECD), the M2, M3, or M4 domains resulted in reduced expression levels and impaired GlyR function including reduced glycine sensitivity and maximal glycine-gated currents (Chung et al., 2013; James et al., 2013).

Here, we investigated three GlyR β variants Y252S, S321F, and A455P from patients with startle disease (Lee et al., 2013; Aboheimed et al., 2019). Mutations have been analyzed using protein biochemical and immunocytochemical analyses, electrophysiological recordings and homology modeling. All three β subunit variants did not show any major trafficking defects. Co-expression with gephyrin resulted in recruitment of GlyR β variants (β^X^) Y252S, S321F, and A455P to gephyrin clusters in transfected HEK-293 cells. At the structural level, Y252S disrupts stacking interactions with surrounding residues of M3 and M4, while S321F most probably stabilizes stacking interactions with other aromatic residues in M3. Functional heteromeric α1β^X^ GlyRs displayed reduced agonist potencies or significantly enhanced maximal chloride ion influx upon activation with glycine thus demonstrating a contribution of the identified β subunit mutations to the startle disease phenotype in affected patients.

## Materials and Methods

### Homology Modeling and Sequence Alignment

For the modeling of the heteromeric αβ glycine receptor, the recent cryo-EM structures of the homomeric α1 subunit GlyR (PBD:6VM0) (Yu et al., 2021) was taken as the template for the pentameric arrangement. The same structure was used for homology modeling of the GlyR β subunit using SWISS-MODEL (Arnold et al., 2006). The stoichiometry of the heteromeric 3α:2β is based on the stoichiometry proposed by Patrizio et al. (2017) to exclude the β-β interface in the heteropentameric arrangement. Mutations in the β subunit were carried in Coot (Emsley and Cowtan, 2004) by taking into consideration the most probable rotamer conformation by considering clashes with the surrounding residues and retaining the geometry of the mutated residue. Structural figures were prepared using ChimeraX (Goddard et al., 2018). Alignments were performed using T-COFFEE web server (Version 11.00, (Notredame et al., 2000; Di Tommaso et al., 2011).

### Molecular Cloning

Full-length cDNAs encoding the human wild-type (WT) or mutated GlyR β variants (β^x^) (with *x* = Y252S, S321F, and A455P) were cloned into the eukaryotic expression vector pRK5 (gift from †P. Seeburg, Heidelberg) and mutagenesis was performed as previously described (James et al., 2013). GlyR β variants were further subcloned into pRK5 harboring a myc-epitope at the N-terminus 5′ of residues ^1^KEKS^4^ representing the first amino acids of the mature GlyR β sequence. eGFP-gephyrin constructs were previously described (Harvey et al., 2004). All expression constructs were fully sequenced to verify successful mutagenesis.

### Cell Lines

HEK-293 cells (Human Embryonic Kidney cells; CRL-1573; ATCC – Global Biosource Center, Manassas, VA, United States) were grown in minimum essential medium (Life Technologies) and COS-7 cells (African Green Monkey Kidney cells; CRL-1651; ATCC – Global Biosource Center, Manassas, VA, United States) were grown in Dulbecco’s modified eagle medium (Life Technologies), both supplemented with 10% fetal bovine serum, L-glutamine (200 mM) and 50 U/ml penicillin and 100 μg/mL streptomycin at 37°C and 5% CO_2_.

### Preparation of Hippocampal Neurons

Neurons were prepared from wild-type CD-1 mice at the embryonic stage 16 (E16). Experiments were approved by the local veterinary authority (Veterinäramt der Stadt Würzburg, Germany) and the Ethics Committee of Animal Experiments, i.e., Regierung von Unterfranken, Würzburg, Germany (license no.: FBVVL 568/200-324/13). Briefly, murine embryos were taken out of the euthanized mother mouse and dissected under a binocular microscope. Hippocampi were dissected out of the embryos and collected in neurobasal medium (21103-049 Life Technologies, Waltham, MA, United States) on ice. Following collection, the tissue was trypsinized using 5 ml of trypsin/EDTA (1 mg/ml) and 50 μl of DNase I (final concentration, 0.1 mg/ml), incubating the suspension at 37°C for 30 min. Trypsinization was stopped with 500 μl of fetal calf serum (final concentration, 10%). After a three-step trituration protocol, the cells were centrifuged at 800 rpm for 15 min. Trituration steps were repeated. Neurons were seeded in 3 cm dishes containing four poly-L-lysine coated coverslips in a density of 150,000 cells per dish. Neurons were grown in neurobasal medium supplemented with 1% 200 mM L-glutamine (25030-024 Life Technologies, Waltham, MA, United States) and 1% B27 (17504-044 Life Technologies, Waltham, MA, United States) with an exchange of 50% medium after 6 days in culture.

### Transfection of Cell Lines and Primary Neurons

#### Transfection of HEK-293 Cells

HEK-293 cells were transiently transfected using a modified calcium-phosphate precipitation method. Transfection was performed at a confluency of 50–75%, 24 h after seeding of 200,000 cells on glass cover slips in 35 mm culture dishes or 1.5 × 10^6^ cells per 10 cm dish. For 35 mm and 10 cm culture dishes GlyR α1, GlyR β or GlyR β variants and eGFP-gephyrin were transfected in a ratio of cDNAs 1:10:5, e.g., 0.2 μg α1, 2 μg β, and 1 μg of eGFP-gephyrin for 35 mm dishes. To label cellular compartments, low copy number vectors were used to exclude effects from overexpression [1 μg; dsRed MEM = fusion of sequence from neuromodulin (GAP-43) and dsRed to label the plasma membrane or dsRed ER = fusion construct of dsRed and calreticulin sequence to label the ER]. The DNA was supplied with 2.5 M CaCl_2_, 0.1x TE buffer and 2x HBS buffer (50 mM HEPES, 12 mM glucose, 10 mM KCl, 280 mM NaCl, 1.5 mM Na_2_HPO_4_) mixed and incubated for 20 min at room temperature. The medium was exchanged after 4–6 h and cells were used for experiments 24–48 h after transfection.

#### Transfection of COS-7 Cells

COS-7 cells were transfected using a DEAE-Dextran transfection protocol. 150,000 cells were seeded on glass cover slips in 35 mm dishes 24 h before transfection. 0.2–2 μg plasmid DNA (same amounts were used as for transfection of HEK-293 cells) in PBS and 10 mg/ml DEAE-Dextran were mixed and added to the cells. After an incubation of 30 min at 37°C, cells were washed and 2 ml culture medium with 10 mM chloroquine were added to the cells. The medium was exchanged again after 2 h and cells were used for immunocytochemical stainings 24–48 h after transfection.

#### Transfection of Hippocampal Neurons

Hippocampal neurons were transfected 3 days after plating using a modified calcium-phosphate precipitation method. 2 μg of DNA (1 μg/μl), 2.5 μl CaCl_2_ (2.5 M), 70 μl Ampuwa water and 25 μl 2xBBS (50 mM BES, 280 mM NaCl, 1.5 mM Na_2_HPO_4_, pH 7.05) were mixed and incubated for 30 min in the dark. Meanwhile, the neuronal medium was aspirated from the hippocampal culture and stored until the end of transfection. Neurons were transfected with the transfection mixture (α1:β in a ratio of 1:5) supplemented by additional 450 μl neurobasal medium for 30 min incubated in the cell culture incubator. The transfection mixture was aspirated, the cells washed twice with Hank’s Balanced Salt Solution (HBSS) medium and flooded with the original neuronal medium. Neurons were used for immunocytochemical staining at DIVs 17–21.

### Immunocytochemical Stainings

Transfected HEK-293, COS-7 cells or hippocampal neurons were fixed using 4% paraformaldehyde with 4% sucrose in phosphate-buffered saline (PBS) for 20 min at room temperature. After washing for three times with PBS, cells were blocked and permeabilized with 5% goat serum with 0.2% Triton-X-100 in PBS for 30 min. Primary antibodies against myc-tagged GlyR β (Synaptic Systems, Göttingen, Germany, 303008, 1:250), Golgi/GM130 (BD Transduction Laboratories, Heidelberg, Germany, 610822, RRID:AB_398141, 1:250), ERGIC-53 (Enzo, ALX-804-602-C100, RRID:AB_2051363, 1:250), gephyrin (Synaptic Systems, Göttingen, Germany, 147111, RRID:AB_887719, 1:100) and synapsin (Synaptic Systems, Göttingen, Germany, 106006, RRID:AB_2622240, 1:500) were diluted in PBS containing 5% normal goat serum and incubated for 1 h, followed by incubation of secondary antibodies goat-α-rabbit-Alexa-488 (Dianova, Hamburg, Germany, 111-546-003, RRID:AB_2338053), goat-α-mouse-Alexa-488 (Dianova Hamburg, Germany, 115-546-003, RRID:AB_2338859), goat-α-mouse-Cy3 (Dianova, Hamburg, Germany, 115-165-003, RRID:AB_2338680), goat-α-rabbit-Cy3 (Dianova, Hamburg, Germany, 111-165-003, RRID:AB_2338000), donkey-α-chicken-Alexa 647 (Dianova, Hamburg, Germany, 703-605-155, RRID:AB_2340379) and goat- α-rabbit-Cy5 (Dianova, Hamburg, Germany, 111-175-006) diluted 1:500 in PBS containing 5% goat serum for 1 h in the dark. After another washing step with PBS, cell nuclei were stained with 4′,6-diamidino-2-phenylindole (DAPI) in PBS for 5 min. Cells were washed again with PBS and ddH_2_O and mounted on a microscope slide in Mowiol.

### Image Analysis

Images of immunocytochemical stainings were captured using an Olympus Fluoview ix1000 microscope with an UPLSAPO 60x oil objective and diode lasers of 405 nm, 495 nm and 550 nm. All images were captured with 1024 × 1024 pixels. For image analysis and processing (eGFP-gephyrin cluster analysis, synapse colocalization, and Western blot quantification) the Fiji/ImageJ Software was used (Schindelin et al., 2012).

### Biotinylation Assay and Immunostaining

Transiently transfected HEK-293 cells co-expressing wild-type GlyR α1β, α1β^x^ variants or α1β together with eGFP-gephyrin were used. 48 h after transfection, medium was removed, and cells were washed three times with ice-cold PBS (GE Healthcare, Freiburg, Germany). The surface proteins were labeled by incubating the cells (10 cm dish) for 30 min with 1 mg/mL EZ-Link Sulfo-NHS-LC-biotin [sulfosuccinimidyl-6-(biotinamido)hexanoate, Pierce Biotechnologies, Rockford, IL, United States], followed by incubation with quenching buffer (192 mM glycine, 25 mM Tris in PBS, pH 8.0) for 10 min. Cells were detached by using ice-cold PBS buffer followed by centrifugation for 10 min at 1,000 × *g*. Cell lysis was performed with TBS (Tris–buffered saline) with 1% Triton-X100 and protease inhibitor mixture tablet (Roche Diagnostics, Mannheim, Germany) and centrifuged for 1 min at 13,000 × *g*. The supernatant (whole protein fraction) was incubated with 50 μl of streptavidin-agarose beads (Pierce Biotechnologies, Rockford, IL, United States) for 2 h at 4°C while rotating. After removing the supernatant, beads were washed three times in TBS buffer. Biotinylated proteins were eluted by boiling with 50 μl of 2x SDS buffer for 5 min at 95°C. 40 μg of surface proteins were analyzed by Western blot.

### SDS-PAGE and Western Blot

Proteins samples were separated by SDS-PAGE using 11% (w/v) gels followed by transfer of separated proteins onto a nitrocellulose membrane (GE Healthcare, Little Chalfont, United Kingdom). After blocking for 1 h with 5% BSA in TBS-T (TBS with 1% v/v Tween 20), membranes were incubated with primary antibodies over night at 4°C (anti-GlyRβ, Synaptic Systems, Göttingen, Germany, 146211, 1:200) or (anti-gephyrin, Synaptic Systems, Göttingen, Germany, 147111, 1:100). Pan-cadherin (Cell Signaling Technology, Danvers, MA, United States, 4068, 1:1,000) or GAPDH (Merck, Darmstadt, Germany, CB 1001, 1:1,000) served as a loading control. Proteins were visualized with the help of horseradish peroxidase (111-036-003 and 115-035-146, Dianova, Hamburg, Germany) and detected through chemiluminescence using clarity^TM^ Western ECL substrate (Clarity Western Peroxide Reagent, Bio-Rad 170-5061, Hercules, CA, United States).

### Electrophysiological Recordings

Electrophysiological characterization was performed on transfected HEK-293 cells using the patch-clamp method for whole cell recordings. Experiments were performed at room temperature. Recording pipettes were pulled from borosilicate capillaries, had an open resistance of 3.5–5.5 MΩ and were filled with internal Buffer [120 mM CsCl, 20 mM N(Et)_4_Cl, 1 mM CaCl_2_, 2 mM MgCl_2_, 11 mM EGTA, 10 mM HEPES; pH 7.2, adjusted with CsOH]. For determination of maximal current amplitudes (I_max_) and EC_50_ values, glycine was applicated in concentrations of 10 μM, 30 μM, 60 μM, 100 μM, 300 μM, 600 μM, 1 mM in external buffer (137 mM NaCl, 5.4 mM KCl, 1.8 mM CaCl_2_, 1 mM MgCl_2_, 5 mM HEPES; pH 7.35, adjusted with NaOH). Glycine concentrations were introduced by an OctaFlow II system (ALA Scientific Instruments, Farmingdale, NY, United States) for 50 ms at 15 PSI. After increasing glycine concentrations, 100 μM picrotoxinin (Sigma-Aldrich, Darmstadt, Germany) + 100 μM glycine in external buffer was applied in the same manner. Current responses were amplified with an EPC-9 amplifier (HEKA Elektronik GmbH, Lambrecht/Pfalz, Germany) and measured at a holding potential of −60 mV using PatchMaster Next software (HEKA Elektronik GmbH, Lambrecht/Pfalz, Germany). Maximal current amplitudes blocked by picrotoxinin of at least 50% of the initial current following glycine application alone was deemed to represent homomeric GlyRs, and were thus excluded from analysis.

### Statistical Analysis

Data were analyzed using Graph Pad Prism or Origin 9 Software and are represented as mean ± SEM (standard error of the mean).

Electrophysiological recordings of transfected cells were performed from at least three different experiments. The numbers of recorded cells are displayed in the figure legends. All other experiments were performed at least three times if not stated elsewhere. Normality of the data was reviewed by Shapiro–Wilk normality test (α = 0.05). Statistical significance was calculated using an unpaired two-tailed Mann–Whitney test or an unpaired *t*-test, depending on the data sets to be analyzed. The χ^2^-test was used for statistical analysis of GlyR subunit compositions during electrophysiological recordings. All *p*-values are given in the section “Results.” The 0-hypothesis was rejected at a level of *p* < 0.05.

## Results

### Novel Glycine Receptor β Mutations Interfere With Stacking Interactions Within the α-Helical Transmembrane Domains and Are Predicted to Affect Protein Stability

**FIGURE 1:**
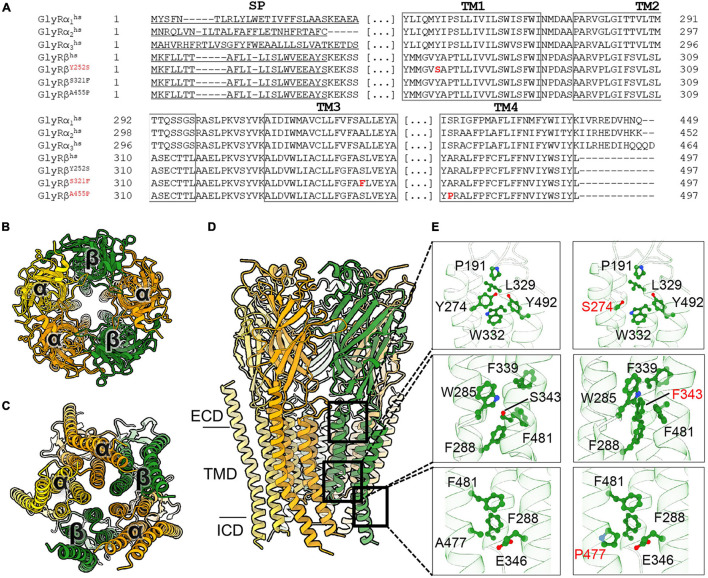
Molecular modeling of novel GlyR β subunit mutants. **(A)** Alignment of GlyR subunits α1, α2, α3, β subunits from human and the β subunit variants concentrating on transmembrane segments. Numbers of amino acid residues in mutant variants refer to non-mature protein, (SP) signal peptide. **(B–D)** Cartoon representation of the 3α:2β glycine receptor heteropentamer homology model viewed from extracellular domain **(B)**, from the intracellular side **(C)** and the membrane plane **(D)**. GlyR α-subunits are colored orange and yellow and β-subunits are colored in green. **(E)** Close-up views of the interaction of residues in WT (left panels) and the mutant (right panels) model. All critical residues are shown as ball and stick, whereas the backbone is shown in cartoon representation, numbering refers to mature protein.

To understand the possible impact of the GlyR β subunit Y252S, S321F, and A455P mutations on the protein structure and functions, we generated a homology model of the 3α:2β heteropentamer (Figure 1). The reported mutations are located in the transmembrane domains M1 to M4 of the GlyR β subunit (Figures 1A–E). In the series of mutations, Y252 (Y252 corresponding to Y274 in the precursor protein including the signal peptide) is located in M1 (Figures 1A,D,E). In the wild-type subunit, this residue is a part of a hydrophobic quadrant with a stacking interaction with W332 from the M3 and van der Waals interactions with Y492 and W493 from M4. In addition, Y252 comes in close proximity to P191, a residue located in the cys-loop of the subunit. Mutation of aromatic tyrosine with hydrophilic serine (Figure 1E), on the one hand might impact the folding of the subunit considering the environment of the residue and also the shorter serine side chain loses the contact to P191 from the cys-loop, which in turn might deregulate the gating properties of the receptor. By contrast, S321 (S321 corresponds to S343 in the precursor) is located in M3 and surrounded by aromatic residues. Mutation of this serine into a bulkier phenylalanine, might in turn favor the hydrophobic environment and provide additional stability into the interactions of M3 with M1 and M4 helices (Figures 1A,D,E). Another mutation resides in a residue located in M4 helix, A455 (A455 corresponding to A477 in the precursor) (Figures 1A,D,E). A mutation in this surface-exposed alanine into proline (Figure 1E) can be speculated not to cause any direct impact on the folding of the subunit, yet the proline might produce a kink in the helix which will in turn compromise the interaction of the subsequent residues in the M4 with its surrounding residues in M1 and M3 helices.

### Trafficking of Glycine Receptor α1β Heteromers to the Cell Surface Is Unaltered for Y252S, S321F, and A455P

**FIGURE 2:**
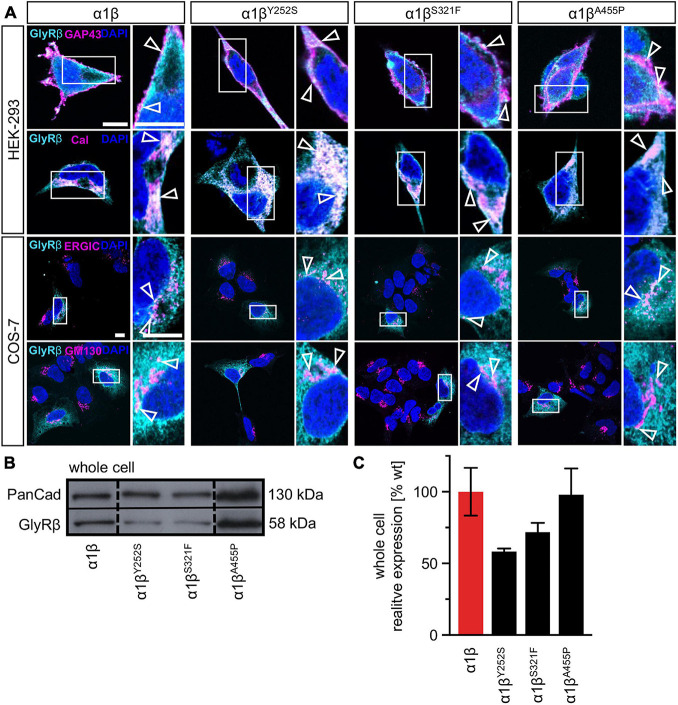
Subcellular expression and trafficking of GlyR β mutants. **(A)** Immunocytochemical staining of HEK-293 (upper two lanes) or COS-7 cells (lower lanes) transfected with GlyR α1 and β WT or β subunit variants (1:10 = α1 WT:β WT or β^x^ variant with x = either Y252S, S321F, or A455P). GlyR β or β^x^ variants were stained with an anti-myc antibody (cyan), cellular compartment marker (GAP-43: cell membrane, calreticulin (Cal): endoplasmic reticulum, ERGIC: ER-Golgi intermediate compartment, GM130: Golgi) are always shown in magenta. Enlargements (marked by white box) are provided on the right next to each image, arrow heads point to co-localization or accumulation of both labeled proteins. Scale bars refer to 10 μm. **(B)** Representative Western blot of whole cell protein lysates from HEK-293 cells transfected with α1 WT and β WT or β^x^ variants in a ratio of 1:2. GlyR β is detected at 58 kDa. Pan-cadherin (Pan-Cad) served as loading control (130 kDa). **(C)** Quantitative analysis of GlyR β and β^x^ variants from whole cell lysates (*n* = 3, three independent experiments) of co-expressed GlyR α1 together with the GlyR β subunit (red bar) or GlyR β^x^ variants (black bars).

The GlyR α1 subunit is transported together with the β subunit to the cellular surface. To investigate an impairment in trafficking, a compartmental analysis was performed in transfected HEK-293 or COS-7 cells. The later were used if the ER-Golgi intermediate compartment (ERGIC) or the *cis-*Golgi (GM130) were stained as COS-7 cells provide a larger cytoplasm. In addition, the ER and the plasma membrane localization were studied. When co-expressed as heteromers, α1β^Y252S^, α1β^S321F^, and α1β^A455P^ exhibited intensive staining in the ER followed by ERGIC and *cis-*Golgi labeling. For all three mutants, membrane staining co-localized with the membrane marker GAP-43 was observed (Figure 2A). Quantitative Western blot analysis from total cell lysates of transfected HEK-293 cells also revealed reduced, but non-significant differences in the whole-cell expression levels between wild-type α1β and the β mutants α1β^Y252S^ 58 ± 2%, α1β^S321F^ 72 ± 7% and α1β^A455P^ 98 ± 18% (Figure 2B and Table 1). No significant reduction of the whole cell protein has also been observed for other startle disease mutations affecting either the α1 or the β subunit (Chung et al., 2013; Atak et al., 2015).

**TABLE 1:** Expression of GlyR β variants in the presence and absence of gephyrin in transfected cells.

GlyR variant	Whole cell GlyR β (%)	*p*-values	*n*	GlyR variant + eGFP gephyrin	whole cell geph (%)	*p*-values	Surface fraction geph (%)	*p-*values	*n*
GlyR α1β	100 ± 17		3	GlyR α1β	100 ± 28		100 ± 23		4
GlyR α1β^Y252S^	58 ± 2	*p* = 0.07	3	GlyR α1β^Y252S^	136 ± 37	*p* = 0.5	191 ± 53	*p* = 0.2	4
GlyR α1β^S321F^	72 ± 7	*p* = 0.2	3	GlyR α1β^S321F^	191 ± 51	*p* = 0.2	190 ± 42	*p* = 0.1	4
GlyR α1β^A455P^	98 ± 18	*p* = 0.9	3	GlyR α1β^A455P^	209 ± 39	*p* = 0.6	168 ± 13	*p = 0.043	4

Significance values: *p < 0.05, n = number of experiments.

### Expression of Heteromeric α1β Glycine Receptors Reveal Altered Ion Channel Function for Y252S, S321F, and A455P

**FIGURE 3:**
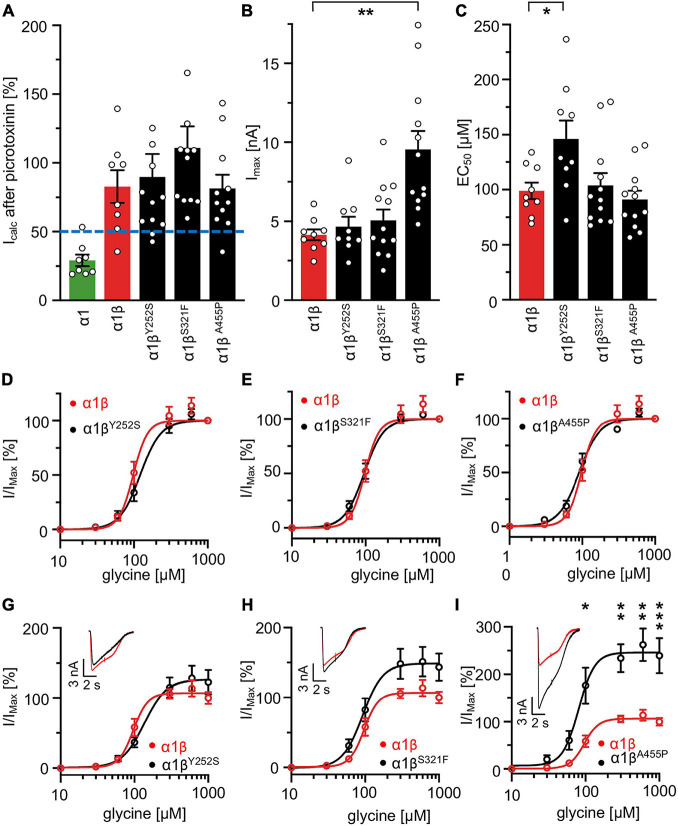
Glycine receptor β subunit mutants alter functional properties of the chloride channel. Electrophysiological measurements of transfected HEK-293 cells. GlyR α1 alone (green bar), co-transfection of α1 and β WT (red bar) or β variants (Y252S, S321F, and A455P; black bars). **(A)** Block of the glycine-gated response using 100 μM glycine and 100 μM of picrotoxinin. Bars show residual currents after picrotoxinin block normalized to currents evoked by 100 μM glycine application. Dotted line shows cut-off for determination of heteromeric receptor configuration. Only cells above the cut-off were used for analysis. **(B,C)** I_max_ and EC_50_ mean values are depicted. **(D–F)** Dose-response curves for α1β (red line) or α1β^x^ variants [Y252S **(D)**, S321F **(E)**, and A455P **(F)**; black lines] using increasing glycine concentrations (10, 30, 60, 100, 300, 600, and 1,000 μM). Values were normalized to the I_max_ mean value of the according α1β variant following application of 1,000 μM glycine. **(G–I)** Dose-response curves of α1β (red line) or α1β^x^ variants [**(G)** Y252S, **(H)** S321F, and **(I)** A455P black lines] normalized to WT α1β I_max_ mean value following application of 1,000 μM glycine. Representative traces at 1,000 μM glycine application of α1β (red traces) and α1β^x^ variants are depicted in the upper left corner of each diagram. Significance values are *p < 0.05, **p < 0.01, and ***p < 0.001.

A reduction of whole cell β subunit does not necessarily equate to reduced glycine-gated chloride ion influx on overexpression of GlyRs in HEK-293 cells. Therefore, we tested the functionality of the heteromeric GlyR α1β^x^ channels. To prove heteromeric receptor expression, picrotoxinin, an GlyR ion channel blocker, was applied together with the agonist (100 μM glycine + 100 μM picrotoxinin) and compared to glycine (100 μM) application alone. If GlyR α1 homomers were measured, the observed block of the glycine-gated response led to a reduction in current amplitude of 29.22% of the original value. By contrast, the observed picrotoxinin block of α1β^x^ channels was in a range of 0–19% meaning residual currents of 81–99% (Figure 3A and Table 2). Thus, cells expressing “heteromeric” GlyRs with a picrotoxinin block larger than 50% were excluded from the analysis, since it was likely that these expressed a significant portion of homomeric α1 subunit GlyRs. At saturating glycine concentrations (600 μM), the maximal current amplitudes for α1β^Y252S^ and α1β^S321F^ were indistinguishable from α1β while α1β^A455P^ exhibited a gain-of-function, as current amplitudes more than doubled in comparison to the α1β WT receptor (9.5 ± 1.2 nA for α1β^A455P^ compared to 4.1 ± 0.4 nA for α1β; *p* = 0.0012; Figure 3B and Table 2). Estimation of the dose-response relationship for the agonist glycine determined a significant increase in the glycine EC_50_ value for the heteromeric receptors α1β^Y252S^ (146 ± 17 μM, *p* = 0.0200; Figures 3C–I and Table 2) and thus a decrease in glycine potency. Mutants α1β^S321F^ and α1β^A455P^ did not result in a change of glycine potency (Figures 3E,F). Normalization of the dose-response curve to the maximal currents obtained from α1β suggested a gain-of-function for α1β^A455P^ (Figures 3G–I).

**TABLE 2:** Electrophysiological properties of GlyR α1β and α1β^x^ variants.

GlyR variant	Glycine EC_50_ (μM)	I_max_ (nA)	I _100__μ__M__p__icrotoxinin +__g__lycine__/_I _100__μ__M__g__lycine_ (%)	*p-*values EC_50_	*p-*values I_m__ax_	*n*
GlyR α1β	99 ± 8	4.1 ± 0.4	90 ± 11			9
GlyR α1β^Y252S^	146 ± 17	4.7 ± 0.6	99 ± 19	*p = 0.02	*p* = 0.47	9
GlyR α1β^S321F^	104 ± 11	5.1 ± 0.7	111 ± 15	*p* = 0.97	*p* = 0.29	12
GlyR α1β^A455P^	91 ± 8	9.5 ± 1.2	86 ± 10	*p* = 0.49	**p = 0.0012	12

Significance values: *p < 0.05; **p < 0.01, n = number of experiments.

### Glycine Receptor β Variants Y252S, S321F, and A455P Are Recruited to Gephyrin Aggregates in Transfected Mammalian Cells

**FIGURE 4:**
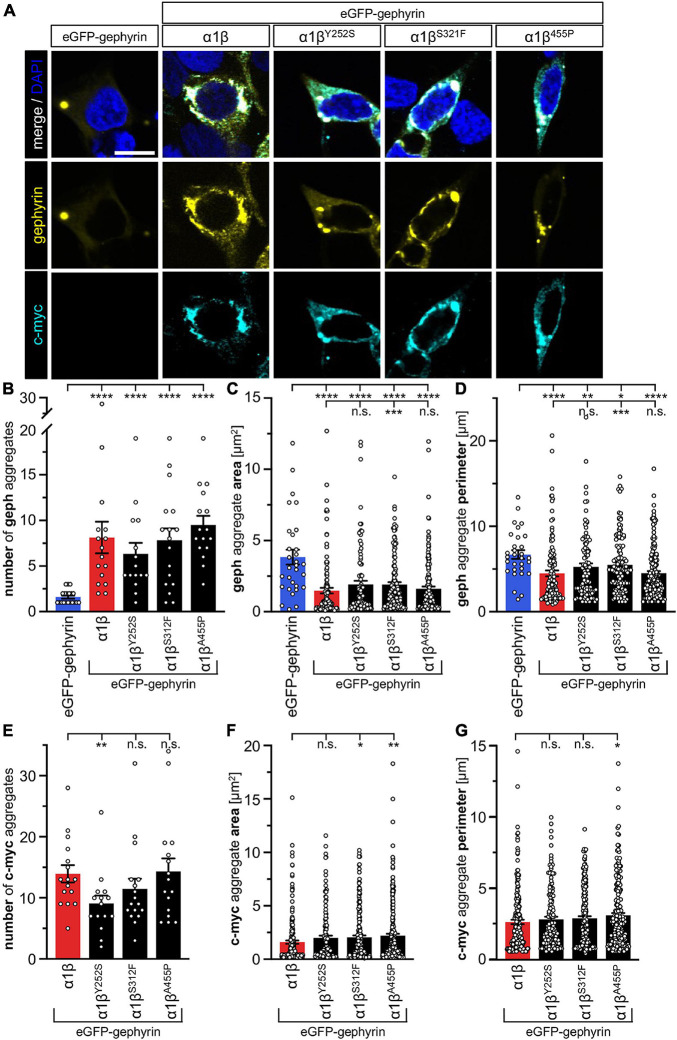
GlyR β variants for cytoplasmic aggregates in the presence of gephyrin. **(A)** Immunocytochemical stainings of HEK-293 cells transfected with eGFP-gephyrin alone or GlyR α1, eGFP-gephyrin and β WT or β variants (Y252S, S321F and A455P; ratio 1:5:10). Myc-tagged GlyR β WT or missense variants were stained with a specific anti-myc antibody (cyan, lower lane), eGFP-gephyrin is shown in yellow (middle lane), merge images are depicted in the upper lane. Nucleus is marked in blue (DAPI). Scale bar refers to 10 μm in all images. **(B–G)** Bar graphs of gephyrin aggregate analysis of HEK-293 cells expressing eGFP-gephyrin (blue bars), eGFP-gephyrin and α1β WT (red bars) or eGFP-gephyrin and α1β variants (black bars): **(B)** number of gephyrin aggregates, **(C)** area of gephyrin aggregates, **(D)** perimeter of gephyrin aggregates, **(E)** number of β (c-myc) aggregates, **(F)** area of β (c-myc) aggregates, **(G)** perimeter of β (c-myc) aggregates, *p < 0.05, **p < 0.01, ***p < 0.001, ****p < 0.0001.

**FIGURE 5:**
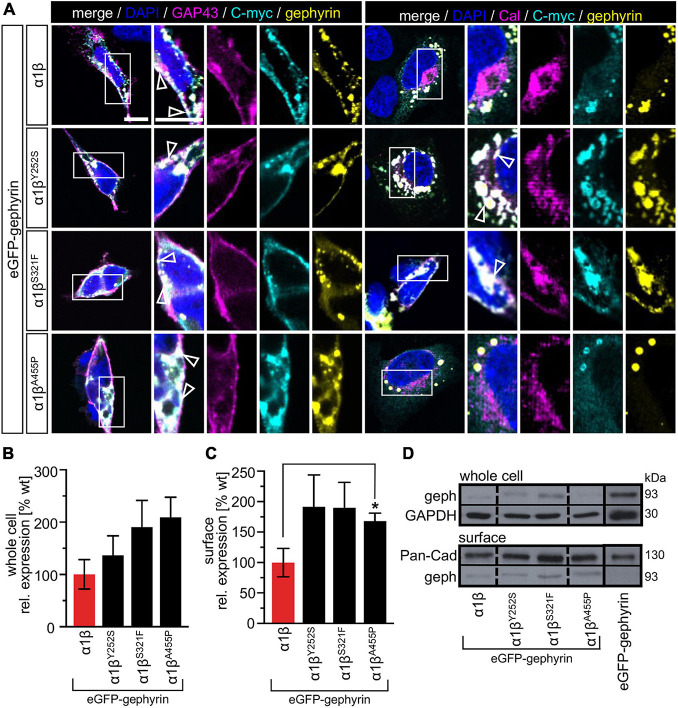
Membrane expression of GlyR β variants is altered in the presence of gephyrin. **(A)** HEK-293 transfected with GlyR α1, eGFP-gephyrin and β WT or β^x^ variants (Y252S, S321F or A455P). Cellular compartment markers (GAP-43: cell membrane, calreticulin (Cal): endoplasmic reticulum) are always co-transfected (magenta). GlyR β WT or variants are stained with an anti-myc antibody (cyan), eGFP-gephyrin is shown in yellow. White boxes mark the areas shown in the enlarged images. Arrow heads point to co-localization or accumulation of eGFP-gephyrin and β WT or β variants. Scale bars refer to 10 μm. **(B,C)** Quantitative analysis of eGFP-gephyrin in whole cell fraction **(B)** and surface **(C)** fractions (*n* = 4, four independent experiments) of co-expressed GlyR α1 together with β WT (red bar) or GlyR β variants (black bars) and eGFP-gephyrin. **(D)** Representative Western blots of whole cell and surface fractions from transfected HEK-293 cells. eGFP-gephyrin (geph) is detected at the appropriate molecular weight of 93 kDa, Pan-cadherin (Pan-Cad) served as loading control for surface fraction (130 kDa) and GAPDH served as loading control for whole cell fraction (30 kDa). Level of significance *p < 0.05.

The GlyR β subunit interacts via a short sequence (R^416^–S^433^) in the M3-M4 intracellular loop with the scaffold protein gephyrin. This interaction is a prerequisite for synaptic localization of the GlyR complex (Meier and Grantyn, 2004). Altered synaptic localization could therefore also underlie a startle disease phenotype at the molecular level. We co-expressed the α1β^x^ combinations together with eGFP-tagged gephyrin (eGFP-gephyrin) in transfected HEK-293 cells. When transfected alone, the construct eGFP-gephyrin forms large highly intense intracellular aggregates (Figure 4A, left panels) (Harvey et al., 2004). Upon co-transfection with the GlyR α1β, these intracellular aggregates are still evident, but were either dispersed or multiple aggregates were formed, arguing for recruitment of the GlyR β subunit to gephyrin accumulations. Interestingly, all β subunit variants Y252S, S321F, and A455P were also recruited to the gephyrin aggregates seen by intense co-localization of eGFP-gephyrin and β subunits (Figure 4A). Gephyrin accumulations were analyzed for number, area and perimeter (Figures 4B–D). The GlyR β WT subunit as well as the β^x^ subunit variants led to significantly enhanced gephyrin aggregate numbers accompanied with decreased aggregate areas in line with recruitment of the GlyR β mutants Y252S, S321F, and A455P together with gephyrin (Figure 4B and Table 3). Similarly, the gephyrin aggregate perimeter was reduced in the presence of α1β and α1β^x^ variants (Figure 4D). A comparison of the gephyrin aggregates between α1β and the β variants revealed a significant increase in the aggregate area and aggregate perimeter for α1β^S321F^ (area: 1911 ± 165 μm^2^, *p* = 0.00037 compared to α1β WT with 1480 ± 195 μm^2^; perimeter: 5503 ± 290 μm, *p* = 0.00059 compared to α1β WT 4497 ± 348 μm, Table 3). In addition to the gephyrin accumulations, the GlyR β subunit was also targeted to intracellular eGFP-gephyrin aggregates in co-expression experiments with the α1 subunit, in contrast to previous immunostainings in the absence of eGFP-gephyrin (Figures 2, 4A). The analysis of the GlyR β subunit aggregation revealed a significantly decreased number for the variant β^Y252S^ (9 ± 1 in comparison to α1β WT 14 ± 1; *p* = 0.0062, Table 3). The β subunit aggregate area was enlarged for mutants β^S321F^ and β^A455P^ (2059 ± 171 μm^2^ with *p* = 0.03 for β^S321F^ or 2202 ± 180 μm^2^ with *p* = 0.0017 for β^A455P^; compared to α1β WT with 1605 ± 151 μm^2^; Table 3) while the perimeter was only significantly increased for β^A455P^ (6234 ± 309 μm, *p* = 0.011 compared to α1β WT 5310 ± 303 μm; Figures 4E–G and Table 3). The observed alterations in number, area and perimeter of the GlyR β^x^ subunit accumulations in eGFP-gephyrin aggregates may have an impact on GlyR biogenesis and finally receptor functionality. The images represent large intracellular GlyR β accumulations with no distinct labeling of the cellular membrane. Membrane expression of the GlyR β variants was revealed in the presence of eGFP-gephyrin and a co-transfected membrane marker (GAP-43). While the GlyR β WT was nicely expressed at the cellular membrane (Figure 5A, upper lane), GlyR β^Y252S^ exhibited both cell surface expression and ER retention (co-localized with calreticulin) and formed large aggregates together with gephyrin. Mutants β^S321F^ and β^A455P^ were also expressed at the membrane but mainly detectable intracellularly (Figure 5A, lower two lanes). We failed to detect the β variants by Western blot. Instead, we used the tight association between GlyR β and gephyrin to measure gephyrin expression in the surface fraction probably still attached to β following a biotinylation assay. Expression of gephyrin alone, did not result in any detectable gephyrin in the membrane fraction (Figure 5D). However, in the presence of the α1β^x^, gephyrin was detectable in both the whole cell and membrane fractions (Figures 5B–D). When co-expressed with α1β^Y252S^ and α1β^S321F^, gephyrin levels in the cell-surface fraction were indistinguishable from the WT α1β level. However, a significant increase of gephyrin was observed when co-expressed with α1β^A455P^ (Figures 5C,D). Thus, although the co-expression with gephyrin led to larger intracellular accumulations of the β^x^ variants, surface expression suggests the formation of functional GlyRs at the cellular membrane.

**TABLE 3:** Quantification of GlyR β and gephyrin accumulations in transfected cells.

**GlyR variant**	**Number of geph aggregates/cell**	***p*-values compared to only gephyrin**	***p-*values compared to GlyR α1β**	***n***	**Number of myc aggregates/cell**	***p-*values compared to GlyR α1β**	***n***

only gephyrin	2 ± 0.2						
GlyR α1β	8 ± 2	****p < 0.0001		16	14 ± 1		16
GlyR α1β^Y252S^	6 ± 1	****p < 0.0001	*p* = 0.5	15	9 ± 1	**p = 0.0062	15
GlyR α1β^S321F^	8 ± 1	****p < 0.0001	*p* = 0.9	17	11 ± 2	*p* = 0.07	17
GlyR α1β^A455P^	10 ± 1	****p < 0.0001	*p* = 0.1	16	14 ± 2	*p* = 0.7	16

**GlyR variant**	**Mean area per geph aggregate (μm^2^)**	***p-*values compared to only gephyrin**	***p-*values compared to GlyR α1β**	***n***	**Mean area per myc aggregate (μm^2^)**	***p*-values compared to GlyR α1β**	***n***

only gephyrin	3837 ± 523						
GlyR α1β	1480 ± 195	****p < 0.0001		16	1605 ± 151		16
GlyR α1β^Y252S^	1925 ± 253	****p < 0.0001	*p* = 0.057	15	1991 ± 215	*p* = 0.1	15
GlyR α1β^S321F^	1911 ± 165	****p < 0.0001	***p = 0.00037	17	2059 ± 171	*p = 0.03	17
GlyR α1β^A455P^	1611 ± 157	****p < 0.0001	*p* = 0.054	16	2202 ± 180	**p = 0.0017	16

**GlyR variant**	**Mean perimeter per geph aggregate (μm)**	***p-*values compared to only gephyrin**	***p-*values compared to GlyR α1β**	***n***	**Mean perimeter per myc aggregates (μm)**	***p-*values compared to GlyR α1β**	***n***

only gephyrin	6703 ± 511						
GlyR α1β	4497 ± 348	****p < 0.0001		16	5310 ± 303		16
GlyR α1β^Y252S^	5245 ± 405	**p = 0.0057	*p* = 0.058	15	5674 ± 372	*p* = 0.3	15
GlyR α1β^S321F^	5503 ± 290	*p = 0.016	***p = 0.00059	17	5814 ± 292	*p* = 0.8	17
GlyR α1β^A455P^	4507 ± 244	****p < 0.0001	*p* = 0.2	16	6234 ± 309	*p = 0.011	16

Significance values: *p < 0.05; **p < 0.01, ***p < 0.001, ****p < 0.0001, n = number of counted cells.

### Interaction With Gephyrin Does Not Further Impact Glycine Receptor Functionality

**FIGURE 6:**
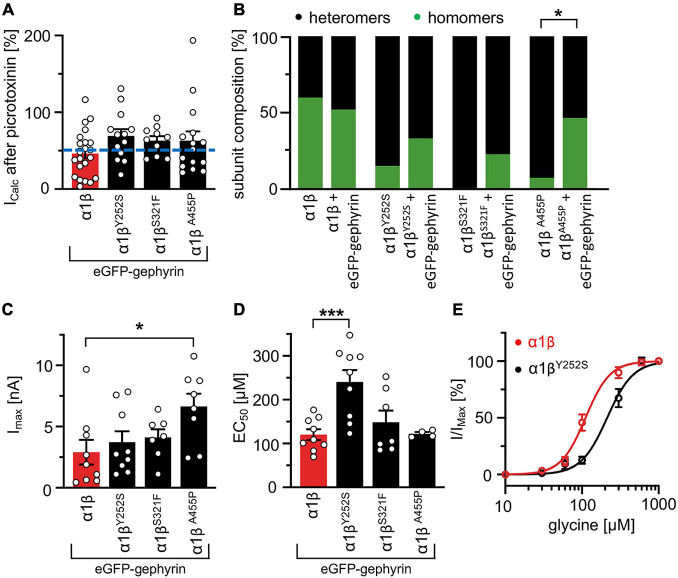
GlyR functional properties do not change in the presence of gephyrin. **(A)** Verification of heteromeric receptor configuration using picrotoxinin block. GlyR α1β WT and α1β^x^ variants were co-expressed with eGFP-gephyrin in HEK-293 cells. The remaining current (%) upon co-application of 100 μM glycine and 100 μM picrotoxinin compared to 100 μM glycine alone is shown. Dotted blue line at 50% picrotoxinin block divides between homomeric (below blue line) and heteromeric (above blue line) receptor configuration. **(B)** Stack plot of the ratio between homomeric (green) and heteromeric (black) receptor configurations of α1β WT and α1β^x^ variants in the absence and presence of eGFP-gephyrin. Significance value *p < 0.05. **(C)** Glycine-activated currents at saturating glycine concentration (1 mM). Note, the GlyR β^A455P^ variant again exhibited significantly increased I_max_ values. **(D)** EC_50_ values for α1β WT and α1β^x^ variants in the presence of eGFP-gephyrin were determined following application of a concentration series of glycine (10, 30, 60, 100, 300, 600, and 1,000 μM). **(E)** Dose response curves of α1β^Y252S^ shows a rightward shift to higher glycine concentration compared to α1β WT in the presence of eGFP-gephyrin. *p < 0.05, ***p < 0.001.

Whole-cell electrophysiology measurements revealed a larger fraction of homomeric α1 GlyRs in cells transfected with α1β and gephyrin compared to cells transfected with α1β alone (Figures 6A,B). Around 40.9% of all cells recorded following transfection of α1β WT or α1β^x^ variants together with eGFP-gephyrin expressed homomeric α1 GlyRs. This is perhaps to be expected given that homomeric α1 subunit GlyRs can escape intracellular “trapping” by intracellular eGFP-gephyrin aggregates. However, clear differences were observed between the WT β subunit and the β^x^ variants in the presence of α1 and gephyrin. While the homomeric α1 receptor portion is similar between α1β WT and α1β WT plus gephyrin, for all three β^x^ variants a larger portion of α1 homomers was detected in the presence of gephyrin (co-expressed α1β^Y252S^ *p* = 0.274; α1β^S321F^ *p* = 0.0565; α1β^A455P^ *p* = 0.0228; Figure 6B). Hence, β^x^ variants appear to preferentially target to intracellular gephyrin aggregates, hindering the formation of heteromeric α1β cell-surface GlyRs. The maximal chloride currents at saturating glycine concentration were again significantly increased for the α1β^A455P^ variant confirming A455P as a gain-of-function mutation (I_max_ 6.6 ± 1.1 nA; compared to α1β with 2.9 ± 1.0 nA, *p* = 0.0270; Figure 6C and Table 4). For α1β^Y252S^, the enhanced EC_50_ value was even more pronounced in the presence of gephyrin (EC_50_ 241 ± 27 μM; compared to α1β with 120 ± 12 μM, *p* = 0.0009; Figures 6D,E and Table 4). Hence, the observed functional alterations for the GlyR β variants were also detectable in a heteromeric complex with GlyR α1 and gephyrin.

**TABLE 4:** Physiological measurements of α1β and α1β^x^ variants in the presence of gephyrin in transfected cells.

GlyR variant	Glycine EC_50_ (μM)	*n*	*p-*values EC_50_	I_max_ (nA)	*n*	*p-*values I_max_	I_picrotoxinin + glycine/_ I_glycine_ (%)	Homomers without gephyrin co-expression (%)	Homomers with gephyrin co-expression (%)	*p-*values	*n*
GlyR α1β	120 ± 12	9		2.9 ± 1.0	9		72 ± 7	60	52	*p* = 0.6	3
GlyR α1β^Y252S^	241 ± 27	9	***p = 0 0009	3.7 ± 0.9	9	*p* = 0.30	84 ± 9	15	33	*p* = 0.274	3
GlyR α1β^S321F^	148 ± 27	7	*p* = 0.32	4.1 ± 0.7	7	*p* = 0.14	73 ± 4	0	23	*p* = 0.0565	3
GlyR α1β^A455P^	122 ± 4	4	*p* = 0.28	6.6 ± 1.1	8	*p = 0.027	92 ± 17	8	47	*p = 0.0228	3

Significance values: *p < 0.05; ***p < 0.001, n = number of experiments.

### Interaction of Glycine Receptor β Subunit Variants With Endogenous Gephyrin and Synaptic Localization in Transfected Primary Neurons

**FIGURE 7:**
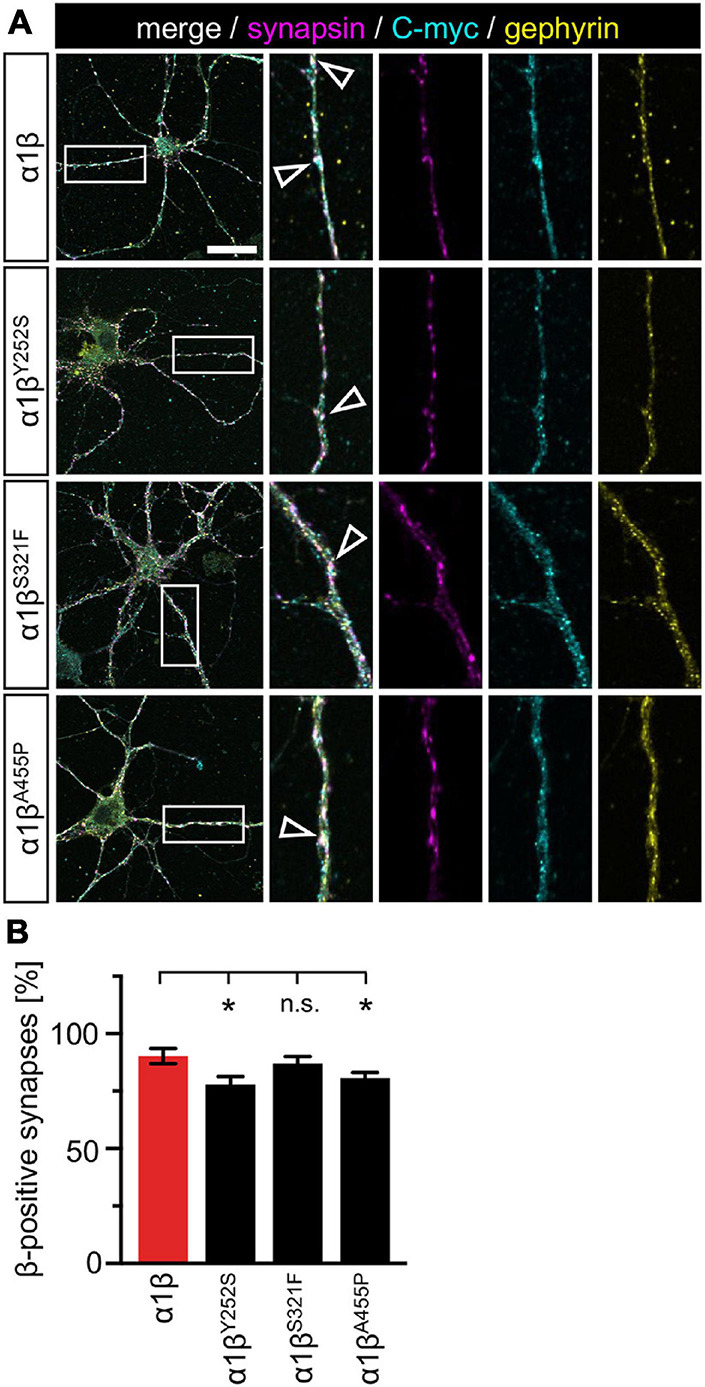
GlyR β variants form clusters with endogenous gephyrin but exhibit less synaptic localization. **(A)** Hippocampal neurons transfected with α1β WT and α1β^x^ variants. The GlyR β subunit was detected with a myc-antibody (cyan), as well as endogenous gephyrin (yellow), and synapsin (magenta). White boxes indicate dendritic areas shown in the enlarged images on the right. White open arrowheads show co-localization of GlyR β in gephyrin-positive and synapsin-positive clusters. Scale bar refers to 25 μm. **(B)** Quantification of GlyR β-positive synapses. Calculation was performed from 40 to 92 dendrites (*n* = 40–92) from transfected neurons obtained from two independent experiments. A ratio of myc-GlyR β-positive synapses versus gephyrin-positive synapses was estimated and is given as β-positive synapses in %. Level of significance *p < 0.05.

The novel GlyR β missense variants still interact with the scaffold protein gephyrin *in vitro* but alter the area or perimeter of intracellular GlyR β-gephyrin accumulations. To assess whether the GlyR-gephyrin interaction is also modified in a synaptic context, hippocampal neurons were transfected with the GlyR α1 and β WT or α1β^x^ variants. Synapsin was used as a marker of synapses. Consistent with previous reports, endogenous gephyrin formed dendritic microclusters. Similarly, the myc-tagged GlyR β subunit and β^x^ variants exhibited clusters along the dendrites of the hippocampal neurons (Figure 7A). However, large intracellular accumulations of GlyR β and gephyrin were not evident. Quantification of gephyrin versus GlyR β at synaptic sites, however, revealed a significant reduction of synaptic β^Y252S^ (*p* = 0.0124) and β^A455P^ (*p* = 0.0208) (Figure 7B and Table 5). Therefore, in addition to the observed impairment of ion channel function, the GlyR β variants β^Y252S^ and β^A455P^ revealed reduced synaptic localization that could contribute to the pathology in individuals with these missense mutations.

**TABLE 5:** Quantification of synaptic localization of GlyR β^x^ variants.

GlyR variant	β-positive synapses (%)	*p-*values	*n*
GlyR α1β	90 ± 3		74
GlyR α1β^Y252S^	87 ± 3	*p = 0.0124	95
GlyR α1β^S321F^	78 ± 3	*p* = 0.9	40
GlyR α1β^A455P^	81 ± 2	*p = 0.0208	85

Significance values: *p < 0.05, n = number of dendrites used for calculation from two independent experiments.

## Discussion

In the present article, we identified functional alterations for the novel GlyR β variants Y252S, S321F, and A455P, that were identified in individuals with startle disease. The most common affected genes are *GLRA1* encoding the GlyR α1 subunit and *SLC6A5* encoding the glycine transporter 2, followed by GLRB encoding the β subunit (Rees et al., 2006; Harvey et al., 2008; Chung et al., 2013; James et al., 2013; Bode and Lynch, 2014). The β subunit is a key component of adult heteromeric α1β GlyRs at synaptic sites (Meier and Grantyn, 2004). Dominant mutations in GlyR α1 mainly affect the M1-M2 domains and the connecting extracellular loop and uncouple agonist binding from channel gating. By contrast, most recessive mutations result in protein truncation, or impair protein trafficking, reducing or eliminating GlyR expression at the cell surface as a consequence (Villmann et al., 2009). The molecular mechanisms underlying GlyR β subunit mutations are less well understood (Chung et al., 2013; James et al., 2013). Studies concentrated on heterologous expression of α1β heteromers in transfected cells. Chung et al., described reduced chloride ion influx rates accompanied by decreased surface expression for GlyR β variants independent of the mutant location in the ECD or transmembrane segments. In addition, James et al. (2013) reported the identification of a spontaneous active channel for the mutation β^L285R^ localized in the M2 domain that caused spontaneous channel opening as well as β^M177R^ (ECD) and β^W310C^ (M3) which resulted in decreased maximal currents. In this study, we assessed three *GLRB* mutations (Y252S, S321F, and A455P) that are all localized in different transmembrane segments: M1, M3, and M4, respectively.

All three β variants Y252S, S321F, and A455P were able to leave the ER, pass the ERGIC (ER-Golgi intermediate compartment), the *cis-*Golgi, and insert into the cellular membrane. We did not detect GlyR β protein accumulations in any of these compartments that could reflect impaired receptor biogenesis or ER-associated degradation (Valkova et al., 2011). Although whole cell GlyR β protein analysis revealed slight decreases in the expression level, the observed differences lacked significance. Efforts to quantitatively estimate the surface expression level of the GlyR β variants failed but immunostainings clearly demonstrated that β subunits co-localized with the membrane marker GAP-43. Therefore, our data are in contrast to most recessive GlyR α1 variants and previously reported β subunit variants that significantly altered receptor trafficking (Chung et al., 2013; Schaefer et al., 2015).

The GlyR β subunit is transported together with the α1 subunit to the cellular surface (Kuhse et al., 1993). Without the α1 subunit, homomeric β subunit GlyRs cannot generate functional ion channels. Previously, it was thought that the β subunit only represented a structural component of the GlyR complex that was important for interactions with gephyrin, so enabling synaptic clustering. However, Grudzinska et al. (2005) identified critical residues in the β subunit located in β sheets β2 (R86) and β7 (E180) that contribute to ligand binding in heteromeric α1β receptors (Grudzinska et al., 2005). The β variants Y252S, S321F, and A455P are not localized in the ECD or close to the proposed residues involved in ligand binding. Missense mutations in a transmembrane segment might, however, impact the steric transduction coupling ligand binding to ion channel opening. Following agonist binding an anticlockwise rotation around the pore axis is initiated thus leading to an anticlockwise rotation of the entire transmembrane domains of all five subunits (Du et al., 2015). The transmembrane domains represent α-helical elements with stacking interactions between aromatic residues localized in the same or adjacent helices (Haeger et al., 2010; James et al., 2013; Tang and Lummis, 2018). The intramembrane aromatic residues are important for pentameric assembly and ion channel function, e.g., allosteric modulation and interaction with lipids (Carswell et al., 2015; Sridhar et al., 2021). The mutation of the aromatic Y252 in the M1 domain into the hydrophilic serine in the GlyR β subunit results in loss of an important interaction to another Y492 in M4 and was predicted to alter ion channel properties. A second GlyR β subunit mutation S321F, results in an aromatic residue substituting for a polar serine. This was predicted to stabilize the M3 structure via an additional interaction with neighboring aromatic ring structures. The third β subunit mutation A455P introduces a kink in the M4 domain, although the overall structure is only marginally affected, flexibility might be altered.

In the GlyR α1 subunit, a tyrosine, is also found at the corresponding amino acid position to GlyR β Y252. A mutation of this tyrosine (Y228C) has been reported in an individual with startle disease, but this has not yet been investigated at the functional level (Forsyth et al., 2007). Other hyperekplexia mutations affecting residues around Y228 in GlyR α1 mainly affected glycine potency (Humeny et al., 2002; Chung et al., 2010; Bode et al., 2013). For the β subunit mutant Y252S, we found increased EC_50_ values suggestive of a reduced glycine potency, while expression levels and maximal currents in response to glycine were unaffected. Mutations of aromatic residues in the M4 domain (W407R in α1 and Y470C in β) found in individuals with startle disease significantly decrease GlyR levels or are retained in the ER (Chung et al., 2013; Schaefer et al., 2015). Hence, data from the GlyR β mutant Y252S illustrate that impaired protein expression is not a general mechanism following disruption of stacking interactions of aromatic residues within or between transmembrane helices.

The GlyR β variant S321F did not exhibit any obvious expression or trafficking deficits and no functional alterations were observed on heterologous expression in HEK-293 cells. This is in line with the structural prediction that this mutant might have a rather stabilizing effect. However, this does not explain why the patient shows characteristic symptoms of hyperekplexia. As this patient also carries a second mutation affecting the splice donor site of intron 4 of the *GLRB* gene, one might assume that the second mutation may significantly contribute to the phenotype in this patient (Lee et al., 2013). Disruption of a splice site can cause aberrant splicing und thus severely affect protein expression level. A reduction to less than 10% of the full-length GlyR β subunit result in typical symptoms of startle disease as observed in the spontaneous mouse model *spastic* (Mulhardt et al., 1994).

The mutant A455P represents a gain-of-function, leaving the EC_50_ unaffected. The increase in glycine efficacy is not accompanied by an increase in surface expressed receptors. Gain of function mutations have also been identified for the GlyR α1 subunit (I43F, Y128C, W170S, Q226E, V280M, and R414H). Increased glycine sensitivity and spontaneous channel activation have been mainly associated with gain-of-function mutations in the GlyR α1 subunit. Clinically, the hyperekplexia phenotype induced by a gain-of-function mutation is not different from a loss-of-function mutation with clonazepam being an effective treatment in many cases (Bode and Lynch, 2014).

*In vivo*, the GlyR complex is anchored at synaptic sites via the β-gephyrin interaction (Kim et al., 2006). The interaction site at the receptor is localized in the large intracellular loop between M3 and M4 (Meyer et al., 1995). Although none of the amino acid substitutions in the β subunit we studied directly affects the interaction site, structural changes in the transmembrane segments might also impact the overall structure and presentation of the gephyrin binding motif. The structure of the M3-M4 intracellular loop is not yet described except for short amino acid sequences following M3 and at the N-terminal end of M4 (Unwin, 2005). The GlyR β mutants localized in transmembrane domains M1, M3, and M4 were investigated in the presence of gephyrin. The observed changes at the functional level were decreased glycine potency for Y252S and an increased glycine efficacy for A455P. These parameters were more pronounced in the presence of gephyrin, while S321F did not display any functional alterations either with or without gephyrin. Interestingly, an increased number of α1 homomers was noticed when the β mutants were co-expressed with the α1 subunit and gephyrin compared to co-expression in the absence of gephyrin arguing for inefficient incorporation of the β subunit mutants into functional receptors in the presence of gephyrin. Gephyrin has been reported earlier to trap α1β receptors in large intracellular aggregates in heterologous expression systems at least (Kins et al., 2000; Grosskreutz et al., 2001).

In the presence of the GlyR α1 and β subunits, gephyrin aggregates increased in number and concomitantly in area and perimeter. We found large cytoplasmic aggregates for the β variants and gephyrin in the cellular cytoplasm with significant increases in area and perimeter for S321F and A455P. These data suggest that inefficient incorporation into functional GlyRs might underlie the pathomechanisms for S321F and A455P. At synapses, inefficient incorporation into the GlyRs would hinder synaptic localization and probably as a consequence inhibitory neurotransmission. Indeed, less GlyR β was found for the functionally impaired variants Y252S and A455P at synaptic sites in transfected hippocampal neurons. Our data strongly suggest that structural changes in the GlyR β subunit have an impact on GlyR β-gephyrin interactions, synaptic localization and thus the intermolecular crosstalk between α and β subunits and gephyrin (Patrizio et al., 2017). As a consequence, GlyR α1 homomers are the favored receptor configuration which are located at extrasynaptic but not synaptic sites. Moreover, aromatic substitutions have a substantial effect on M4/M1-M3 interactions and interactions with surrounding lipids, also altering ion channel function (Haeger et al., 2010; Carswell et al., 2015; Henault et al., 2019; Sridhar et al., 2021).

In summary, the novel GlyR β subunit mutations result in loss- or gain-of-function and/or impaired synaptic GlyR clustering due to altered interactions between GlyR β and gephyrin. Together, our data provide novel insights into the contribution of the GlyR β subunit to startle disease pathology.

## Data Availability Statement

The original contributions presented in the study are included in the article/supplementary material, further inquiries can be directed to the corresponding author.

## Ethics Statement

The animal study was reviewed and approved by the local veterinary authority (Veterinäramt der Stadt Würzburg) and Committee on the Ethics of Animal Experiments, i.e., Regierung von Unterfranken, Würzburg (License number FBVVL 568/200-324/13).

## Author Contributions

CV, NS, RH, and CS participated in research design. IP, A-LE, NS, RH, CV, and VK conducted the experiments. IP, A-LE, NS, and VK performed the data analysis. CV, RH, and NS wrote the manuscript. All authors contributed to the article and approved the submitted version.

## Conflict of Interest

The authors declare that the research was conducted in the absence of any commercial or financial relationships that could be construed as a potential conflict of interest.

## Publisher’s Note

All claims expressed in this article are solely those of the authors and do not necessarily represent those of their affiliated organizations, or those of the publisher, the editors and the reviewers. Any product that may be evaluated in this article, or claim that may be made by its manufacturer, is not guaranteed or endorsed by the publisher.
